# Metformin and the ATM DNA damage response (DDR): Accelerating the onset of stress-induced senescence to boost protection against cancer

**DOI:** 10.18632/aging.100407

**Published:** 2011-12-12

**Authors:** Javier A. Menendez, Sílvia Cufí, Cristina Oliveras-Ferraros, Begoña Martin-Castillo, Jorge Joven, Luciano Vellon, Alejandro Vazquez-Martin

**Affiliations:** ^1^ Translational Research Laboratory, Catalan Institute of Oncology, Girona, Catalonia, Spain; ^2^ Girona Biomedical Research Institute, Girona, Catalonia, Spain; ^3^ Unit of Clinical Research, Catalan Institute of Oncology, Girona, Catalonia, Spain; ^4^ Centre de Recerca Biomèdica, Hospital Universitari Sant Joan de Reus, Institut d'Investigaciò Sanitària Pere Virgili, Universitat Rovira i Virgili, Reus, Catalonia, Spain; ^5^ Fundación INBIOMED, Cell Reprogramming Unit, San Sebastián Basque, Country, Spain

**Keywords:** Metformin, cancer, ATM, AMPK, genome stability, senescence, autophagy, aging

## Abstract

By activating the ataxia telangiectasia mutated (ATM)-mediated DNA Damage Response (DDR), the AMPK agonist metformin might sensitize cells against further damage, thus mimicking the precancerous stimulus that induces an intrinsic barrier against carcinogenesis. Herein, we present the new hypothesis that metformin might function as a *tissue sweeper* of pre-malignant cells before they gain stem cell/tumor initiating properties. Because enhanced glycolysis (the Warburg effect) plays a causal role in the gain of stem-like properties of tumor-initiating cells by protecting them from the pro-senescent effects of mitochondrial respiration-induced oxidative stress, metformin's ability to disrupt the glycolytic metabotype may generate a cellular phenotype that is metabolically protected against immortalization. The bioenergetic crisis imposed by metformin, which may involve enhanced mitochondrial biogenesis and oxidative stress, can lower the threshold for cellular senescence by pre-activating an ATM-dependent *pseudo*-DDR. This allows an accelerated onset of cellular senescence in response to additional oncogenic stresses. By pushing cancer cells to use oxidative phosphorylation instead of glycolysis, metformin can rescue cell surface major histocompatibility complex class I (MHC-I) expression that is downregulated by oncogenic transformation, a crucial adaptation of tumor cells to avoid the adaptive immune response by cytotoxic T-lymphocytes (CTLs). Aside from restoration of tumor immunosurveillance at the cell-autonomous level, metformin can activate a senescence-associated secretory phenotype (SASP) to reinforce senescence growth arrest, which might trigger an immune-mediated clearance of the senescent cells in a non-cell-autonomous manner. By diminishing the probability of escape from the senescence anti-tumor barrier, the net effect of metformin should be a significant decrease in the accumulation of dysfunctional, pre-malignant cells in tissues, including those with the ability to initiate tumors. As life-long or late-life removal of senescent cells has been shown to prevent or delay the onset or progression of age-related disorders, the *tissue sweeper* function of metformin may inhibit the malignant/metastatic progression of pre-malignant/senescent tumor cells and increase the human lifespan.

The ability of our cells to maintain genomic integrity is fundamental for adequate protection from developmental defects, cancer, and aging. Central to this process is the ability of cells to accurately recognize and correctly repair DNA damage in a timely manner to then allow regulated and orderly progression through the cell cycle. By linking genome stability surveillance, cell cycle, and energy metabolism, the ~370 kDa Serine (Ser)/Threonine (Thr) kinase ataxia telangiectasia mutated (ATM) has begun to emerge as a central DNA damage checkpoint that connects cellular bioenergetics with cancer predisposition and aging [1-4]. ATM is a member of the phosphoinositide 3-kinase-related protein kinase (PIKK) family to which the master regulator of cell growth and metabolism mammalian target of Rapamycin (mTOR) also belongs [5]. ATM is a well-known primary regulator of the cellular response to DNA double-strand breaks (DSBs). A number of studies have recently established that oxidative stress also activates ATM, even in the absence of DSBs. In response to oxidative stress, ATM is phosphorylated at Ser-1981, which results in phosphorylation of its substrates, including p53, the master controller of DNA metabolic stresses, and AMP-activated protein kinase-α (AMPKα), the key sensor of fuel and energy status [6, 7].

## ATM: Connecting the energy restriction mimetic metformin to its metabolic target AMPK

Energetic stress due to glucose restriction increases the AMP/ATP ratio. Treatments with drugs that increase the AMP/ATP ratio, including the AMP analog 5-aminoimidazole-4-carboxamide-1-β-ribofuranoside (AICAR) or the anti-diabetic biguanide metformin, activate AMPKα through phosphorylation of Thr-172 and also increase the levels of the AMPKα protein. Although several proteins can phosphorylate AMPKα (e.g., the master upstream Ser/Thr kinase 11 (STK11)/Liver Kinase B1 [LKB1]), it should be noted that activating phosphorylation of AMPKα in response to energetic stress takes place in an ATM-dependent and STK11/LKB1-independent manner [7]. Accordingly, the selective ATM inhibitor KU-55933 markedly reduces the AMPKα-activating effects of metformin in rat hepatoma cells, functionally supporting the first genome-wide association study that unexpectedly found the *ATM* gene as the causal modulator of glycemic responsiveness to metformin among type 2 diabetic patients [8]. Indeed, treatment with the ATM inhibitor KU-55933 is sufficient to prevent metformin-induced phosphorylation of AMPKα and of the AMPKα downstream target Acetyl-CoA Carboxylase (ACC), concluding that ATM works upstream of AMPKα and that ATM is required for a full response to metformin [8]. Although these results support and extend previous reports of ATM involvement in the activation of AMPKα by stimuli other than metformin [7, 9, 10], metformin's ability to function as a general activator of the ATM-dependent DDR pathway remains to be explored to prove a causal link between the metformin-induced activation of ATM and the diminished risk of developing cancer in individuals taking this drug [11].

We have recently added metformin to the growing list of agents that may have potent cancer-preventive properties by activating the ATM-regulated DDR pathway [12]. The treatment of cultured tumor cells with millimolar concentrations of metformin was found to promote significant activation of ATM, as determined by immunofluorescence microscopy using a monoclonal antibody directed against Ser-1981-phosphorylated ATM. Because cellular DNA damage and particularly the induction of DSBs result in activating phosphorylation of ATM at Ser-1981 and Histone H2AX at Ser-139, we also explored whether the Ser-139 Histone H2AX phosphorylation was altered in response to metformin. Metformin-induced induction of phospho-γH2AX^Ser139^ foci was not accompanied by the expected incorporation of 53BP1 to nuclear repair foci, and metformin-induced Ser-1981 ATM phosphorylation displayed a uniform, nuclear signal that failed to colocalize with phospho-γH2AX^Ser139^ foci. Thus, we termed these metformin-triggered events "*pseudo-DDR"* [13] to distinguish them from a *bona fide* DDR triggered in response to *true* DNA damage. Importantly, “metformin-induced *pseudo-DDR*” was accompanied by the activation of functional elements typically involved in ATM-regulated genomic stress. First, metformin treatment greatly enhanced phosphorylation of Chk2 at Thr-68, an ATM kinase-dependent event that mediates the response of the ATM pathway following DNA damage [14, 15]. Second, metformin exposure notably enhanced phosphorylation of cAMP-Response Element Binding protein (CREB) at Ser-133, an ATM kinase-regulated event in response to oxidative DNA damage and DNA replication stress [16, 17].

## Metformin and ATM-sensed energy metabolism: Reactivating oxidative phosphorylation biogenesis to impede glycolytic cancer cell growth

As metformin is thought to activate AMPK by inhibiting oxidative phosphorylation [18, 19] and because phosphorylation of CREB at Ser-133 can be observed in cultured cells that have been incubated with oxidative phosphorylation inhibitors [20], it could be argued that metformin-induced CREB activation might merely reflect metformin's ability to impair mitochondrial activity in tumor cells. However, CREB phosphorylation is pivotal in mediating peroxisome proliferator-activated receptor gamma coactivator-1alpha (PGC-1α)-stimulated mitochondrial biogenesis [21-24]. Therefore, metformin-stimulated phosphorylation of CREB at Ser-133, which activates the promoter of PGC-1α and increases PGC-α mRNA and protein expression [25, 26], can also be viewed as part of the mechanism through which metformin may control mitochondrial biogenesis in tumor cells. Tumor cells are dependent on glycolysis to support their metabolic requirements; even under aerobic conditions, tumor cells continue to rely on glycolysis rather than oxidative phosphorylation (Warburg effect), resulting in high glucose requirements to generate energy and biosynthetic precursors because of the increased availability of glycolytic intermediates [27-30]. As such, metformin-induced reactivation of oxidative phosphorylation biogenesis may contribute to the growth arrest of cancer cells. A recently developed high-throughput respirometric assay for mitochondrial biogenesis used the Seahorse Bioscience analyzer to measure mitochondrial function in real time. In adapted primary cultures of non-glycolytic renal proximal tubular cells, metformin augmented mitochondrial biogenesis [31]. Recent experiments from our own laboratory have established that culturing human cancer cells in the presence of metformin significantly enhances the expression of cytochrome c oxidase I (COX-1) and mitochondrial succinate dehydrogenase (SDH-A), which are encoded by mitochondrial and nuclear genomes, respectively (Oliveras-Ferraros C, Cufí S, Vazquez-Martin A, Menendez OJ, Martin-Castillo B, Joven J, Menendez JA. Metformin rescues cell surface major histocompatibility complex class I deficiency caused by oncogenic transformation. *Submitted for publication*). Using cancer cell lines, non-cancer cells, embryonic cells and Rho(0) cells (i.e., cells depleted of mitochondrial DNA), Jose et al. [32] recently confirmed that the AMPK agonist AICAR exhibits a strong and cancer-specific growth effect that depends on the bioenergetic signature of the cells and involves upregulation of oxidative phosphorylation. In fact, the sensitivity to pharmacological activation of AMPK is higher when cells display a high proliferation rate accompanied by a low steady-state content of ATP. Although it remains to be established if AMPKα-related induction of mitochondrial biogenesis to increase oxidative phosphorylation is instrumental and possibly required for the anti-cancer/anti-aging effects of metformin [33, 34], it is becoming clear that some health-promoting capabilities of metformin may rely on its ability to function as a *bona fide* glucose-starvation mimetic. Accordingly, recent experiments from our own laboratory have confirmed that, when added to glucose-free medium, where growth is highly oxidative phosphorylation-dependent, metformin drastically increases apoptotic cell death in glucose-addicted cancer cell cultures. Because transformed human cell types appear to be more sensitive to glucose deprivation-induced cytotoxicity and metabolic oxidative stress than non-transformed human cell types, we suggest that a rational use of metformin in combination with fasting could significantly potentiate the effects of chemotherapy in cancer while protecting normal cells, thus further increasing the therapeutic window [35-38] (Oliveras-Ferraros C, Cufí S, Vazquez-Martin A, Menendez OJ, Joven J, Martin-Castillo B, Menendez JA. Glucose deprivation enhances metformin-induced apoptosis in a breast cancer cell type-dependent manner: Implications for cyclotherapy. *Manuscript in preparation*).

In the last issue of *Aging*, Halicka et al. [39] reported that treatment of normal mitogenically stimulated lymphocytes or tumor cell lines treated with metformin attenuated ATM activation and constitutive H2AX phosphorylation. Their observation that cells treated with metformin have reduced expression of Ser-1981-phosphorylated ATM and Ser-139-phosphorylated Histone H2AX is in contrast not only to data previously reported by our own group in *Cell Cycle* [12] but also to those presented by Dian et al. [40] in a recent issue of *PLoS One*. Using human diploid fibroblasts (HDFs), these authors reported that treatment with pharmacological agents increasing the AMP/ATP ratio (i.e., AICAR and metformin) is molecularly equivalent to the effects of glucose restriction in terms of activation of the ATM/AMPK pathway. On one hand, glucose restriction-induced activation of AMPK-driven intracellular signaling was found to be an ATM-dependent process. Thus, the ability of glucose restriction to increase the activating phosphorylation of AMPKα cannot be observed in ataxia-telangiectasia (A-T) cells. On the other hand, treatment of HDFs with the AMPK agonists AICAR or metformin activates ATM at Ser-1981, increases the overall levels of ATM protein and activates AMPK [40]. These findings together indicate that the energetic stress that is induced by glucose restriction or metformin treatment can activate the ATM/AMPKα pathway to induce autophagy and likely cellular senescence. These observations are consistent with the idea that disruption of an energetic stress-induced checkpoint through the loss of ATM function may provide a growth advantage to cells under energetic stress but exacerbate cytotoxic responses to metformin [41].

**Figure 1:**
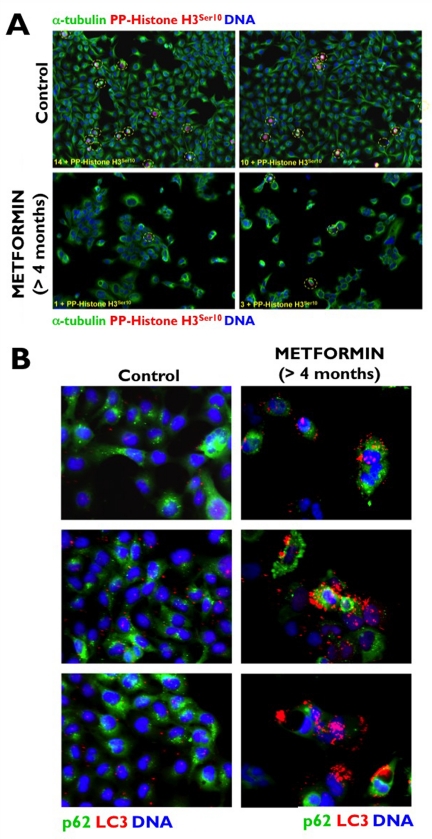
Chronic exposure to metformin suppresses the proliferative activity of human [A431] cancer cells (**A**) to enter a form of permanent cell-cycle arrest accompanied by the induction of “self-digesting” autophagy (**B**).

**Figure 2:**
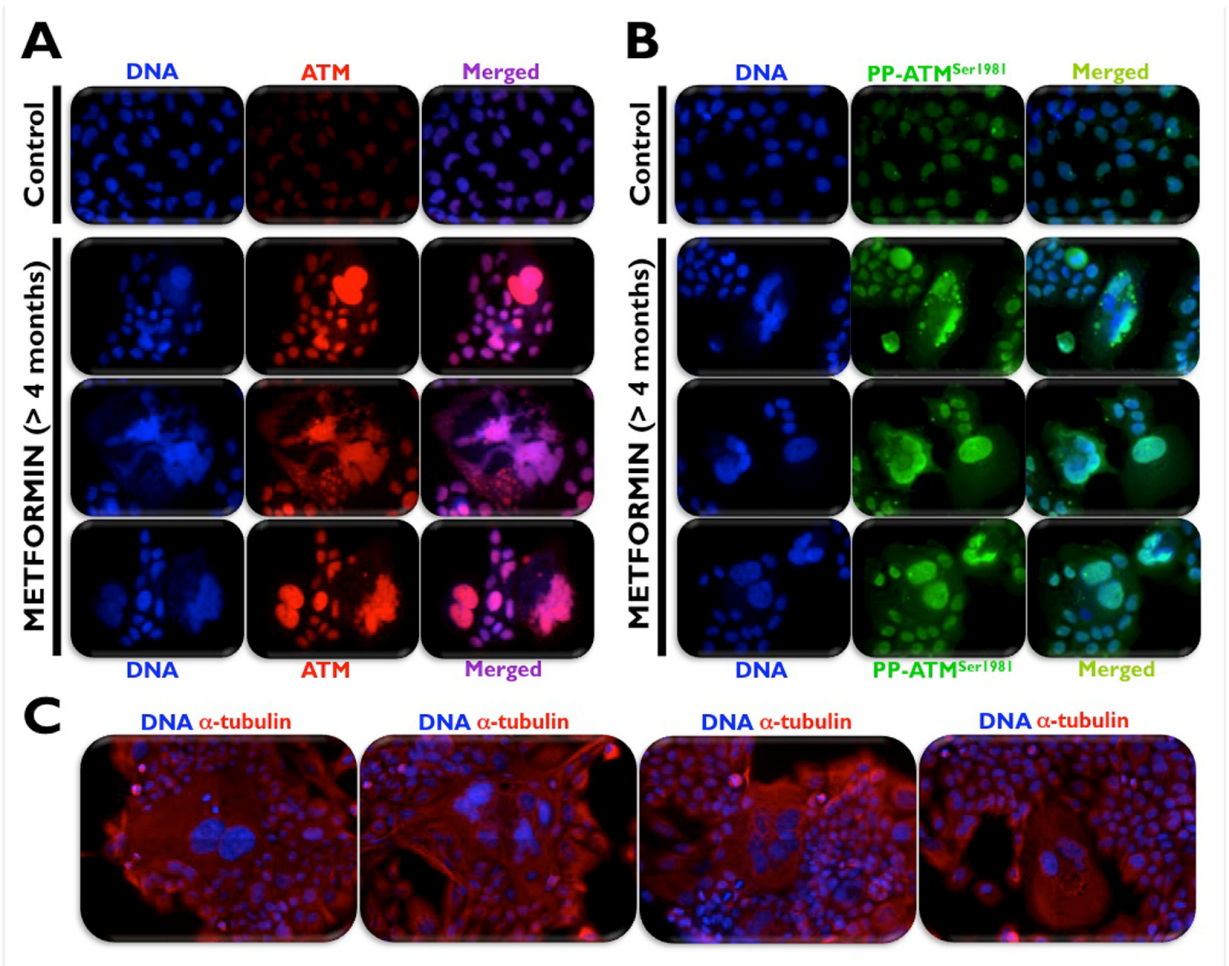
Chronic exposure to metformin activates the DNA damage sensor kinase ATM in poly-morphic, polyploidy [A431] cancer cells that contained giant nuclei and/or multiple varied size nuclei, including micronuclei (**A**, **B**). Chronically-treated cells demonstrated a large, spreading and flattening morphology, typical of senescent cells (**C**).

We recently hypothesized that the unexpected ability of metformin to promote the activation of ATM may be due to the short (24 or 48 h) time courses of most published studies on metformin-induced energetic stress and human cancer cell death *in vitro*. To test this hypothesis and to simulate patients receiving metformin on a daily basis, we maintained A431 epidermoid cancer cells in long-term uninterrupted subculture with metformin concentrations as high as 10 mmol/L for longer than 4 months before starting any experimental procedure. Metformin-induced loss of proliferative potential, as measured by the absence of immuno-reactive Histone H3 phosphorylated at Ser-10 (Figure 1A), was accompanied by chronic activation of autophagy, as measured by confocal imaging of the recruitment of ATG8/LC3 to autophagic vesicles (“LC3 puncta”) and loss of the specific autophagy receptor p62/SQSTM1, a protein that is selectively degraded by autophagy (Figure 1B) [42]. Of note, growth retardation and subsequent arrest of A431 tumor cells in response to the chronic energetic stress imposed by continuous exposure to metformin drastically up-regulated ATM activity and ATM protein accumulation. Indeed, fluorescence microscopic analyses revealed a massive accumulation of a uniform, nuclear signal of both total ATM (Figure 2A) and Ser-1981 phosphorylated ATM (Figure 2B). Furthermore, A431 cells chronically treated with metformin displayed flattened, giant, polynucleated morphology (Figure 2C). These findings not only reaffirm our earlier results and those reported by Duan et al. [40] on metformin-induced activation of the ATM/AMPKα pathway, but they additionally suggest that metformin-mimicked glucose restriction appears to reactivate the senescence program in cancer cells (Cufí S, Vazquez-Martin A, Oliveras-Ferraros C, Martin-Castillo B, Vellon L, Menendez JA. Metformin lowers the threshold for stress-induced cellular senescence. *Manuscript in preparation*).

The above mentioned data suggest an attractive complementary strategy by which we might prevent or halt cancer that is functionally compatible with activation of the ATM pathway: the senescence process is dependent on ATM signaling, and senescence can be bypassed or suppressed by microinjection of kinase-dead constructs of ATM or by treatment with ATM inhibitors [42-46]. Metformin-treated A431 cell cultures typically revealed senescent cells with a damaged nucleus that, in some cases, appeared to evolve through progressive encircling of the nucleus by refringent components. Numerous dense particles (probably damaged components) and several autophagic vacuole-like structures of different sizes were observed within large cells displaying multiple nuclei, micronucleation, or lobulated nuclei. Chronic exposure of cancer cells to metformin might provoke permanent *senescent-cell growth arrest* as a result of a high macroautophagic activity that continuously targets many vital metabolic cancer cell components. This supports earlier studies demonstrating that, in contrast to the widely accepted antioxidant properties of the anti-aging polyphenol resveratrol, chronic culture of cancer cells with resveratrol initiates replication stress via activation of the ATM pathway and induces senescence associated with mitochondria-increased reactive oxygen species (ROS) levels [47]. This report and our current findings appear to functionally link the cell cycle with pro-oxidant/pro-senescent effects of anti-aging compounds in cancer cells [39]. In contrast, Halicka et al. found that the anti-oxidant activity of metformin is functionally linked to enhanced genomic stability, the pivotal mode of action underlying the anti-aging effects of metformin. At present, we cannot explain the apparent discrepancy of our results [12] and those of Duan et al. [40] with the data presented by Halicka et al. [39]. These contradictory hypotheses must be tested adequately before concluding the ultimate mechanism by which metformin exerts anti-cancer and/or anti-aging effects.

### Metformin accelerates the onset of cellular senescence in human diploid fibroblasts (HDFs)

The onset of cellular senescence is thought to protect against the initiation of tumor formation in response to certain cellular stresses, including genotoxic and energetic stresses [48]. Environmental factors that place oxidative stress on cells promote the early onset of cellular senescence by significantly increasing the AMP/ATP ratio and activating stress pathways involving AMPK [49]. AMP/ATP ratios are significantly higher in senescent fibroblasts compared with young fibroblasts and, accordingly, *in vitro* senescence is accompanied by a marked elevation of AMPK activity. Indeed, ATP depletion in senescent fibroblasts is due to the dysregulation of glycolytic enzymes and a failure to maintain ATP levels, which finally leads to a drastic increase in cellular AMP. This, in turn, acts as a growth-suppressive signal that induces premature senescence [50]. Within this model, escaping fromcellular senescence and becoming immortal constitutes a crucial step in oncogenesis that most tumors require for ongoing proliferation [51].

The cumulative oxidative damage induced by growth in conditions that are hyperoxic (by the standard of living tissues) leads to the onset of senescence in HDFs and mouse embryo fibroblasts (MEFs). Indeed, when HDFs/MEFs are propagated in hypoxic conditions (1-3%) rather than the commonly used 20% oxygen, HDFs/MEFs avoid senescence; when grown in 20% oxygen, HDFs/MEFs rapidly accumulate DNA damage and eventually initiate a positive feedback loop of oxidative damage and growth arrest that masquerades as cellular senescence [51, 52]. Immortalized MEFs and mouse/human embryonic stem cells display higher glycolytic flux with reduced oxygen consumption and therefore present more resistance to oxidative damage than senescent cells. As such, they demonstrate the Warburg effect (enhanced glycolysis), which plays a causative role in cell immortality by protecting cells from senescence induced by oxidative damage [53-56]. Thus, it can be speculated that exogenous supplementation with metformin should increase the population-doubling potential of cultured HDFs and MEFs by preventing the accumulation of ROS and oxidative damage as suggested by Halicka et al. [39].

**Figure 3:**
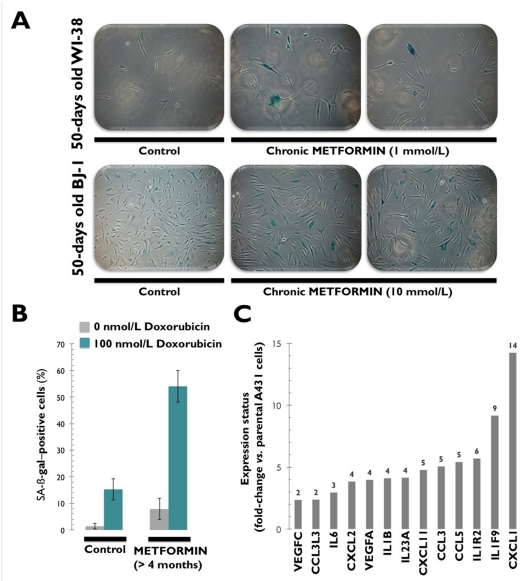
Chronic exposure to metformin accelerates the onset of replicative senescence in human [WI-38 and BJ-1] fibroblast cultures confirmed by senescence-associated β-galactosidase (SA-β-gal) staining (**A**). Chronic exposure to metformin sensitizes [MCF-7] cancer cells to the senescence program activated by the DNA-damaging drug doxorubicin (**B**). Chronic exposure to metformin transcriptionally activates a senescence-associated secretory phenotype (SASP) or senescence messaging secretome (SMS) involving the production of factors that reinforce the senescence arrest, alter the surrounding microenvironment, and trigger immune surveillance of the senescent cells.

Conversely, recent experiments conducted in our laboratory have concluded that chronic exposure to millimolar concentrations of metformin (1 and 10 mmol/L) drastically reduces the lifespan of non-transformed HDFs by accelerating replicative cellular senescence (Figure 3A). Indeed, metformin exposure reduced cumulative population doublings by up to 70% in HDFs (Cufí S, Vazquez-Martin A, Oliveras-Ferraros C, Martin-Castillo B, Vellon L, Menendez JA. Metformin lowers the threshold for stress-induced cellular senescence. *Manuscript in preparation*). Metformin's ability to accelerate the onset of replicative senescence was more significant in WI-38 fetal lung HDFs, which are highly sensitive to stress-induced pre- mature senescence [57]. BJ-1 fibroblasts required longer exposures to higher concentrations of metformin, as they are extremely resistant to hyperoxia and H_2_O_2_ [58, 59]. Although we did not explore the activation status of ATM in HDFs chronically exposed to metformin, it is reasonable to conclude that the metformin-lowered threshold for stress-induced senescence must be explained in terms of metformin-augmented oxidative damage in HDFs. In other words, metformin-accelerated replicative senescence might mostly rely on metformin's ability to establish a stronger DDR-dependent cell cycle arrest because exogenous supplementation with metformin appears to synergistically enhance hyperoxic culture-induced DNA damage and cellular senescence in cultured HDFs.

### 2. Metformin sensitizes HDFs and cancer cells to DSB-induced cellular senescence

Doxorubicin is an anthracycline that indirectly causes DSBs, activates ATM-dependent signaling, and induces cell senescence at concentrations significantly lower than those required to induce apoptotic cell death. Treatment of cells with doxorubicin leads to the phosphorylation of Histone H2AX on Ser-139, with dependence on ATM for the initial response [60]. Treatment with doxorubicin also stimulates ATM autophosphorylation on Ser-1981 and the ATM-dependent phosphorylation of numerous effectors in the ATM-signaling pathway, including Chk2, in a ROS-dependent manner [60]. Because free radical scavengers have been shown to attenuate the accelerated senescence response triggered by treatment with a low concentration of doxorubicin in MCF-7 breast cancer cells [61-63], Halicka's hypothesis that metformin acts as an anti-oxidant that enhances genome stability via ATM inhibition [39] would dictate that metformin treatment must efficiently block doxorubicin-induced senescence [64]. We recently assessed whether metformin treatment can regulate the senescence-like growth arrest induced by doxorubicin in primary MEFs from wild-type (p53^+/+^) mice. Of note, exposure of MEFs to millimolar concentrations of metformin (1 and 10 mmol/L) augmented baseline senescence in doxorubicin-untreated control cultures and notably potentiated cell senescence triggered by doxorubicin-induced DNA damage (Cufí S, Vazquez-Martin A, Oliveras-Ferraros C, Martin-Castillo B, Vellon L, Menendez JA. Metformin lowers the threshold for stress-induced cellular senescence. *Manuscript in preparation*). Furthermore, we maintained MCF-7 breast cancer cells (wild-type p53) in long-term uninterrupted subculture with metformin concentrations as high as 10 mmol/L for longer than 4 months and then challenged them with a senescence-inducing concentration of doxorubicin. Interestingly, the pre-conditioned MCF-7 cells became sensitized to senescence induction by low doses of doxorubicin (Figure 3B). We observed that sequential incubation with metformin, followed by 100 nmol/L of doxorubicin, produced a drastic change in the cellular response program. In response to doxorubicin-induced stress, wild-type MCF-7 cells showed low levels of SA-β-gal positive cells (~15%), and MCF-7/Metformin cells showed very high levels (~54%). This indicated a senescent-like phenotype without signs of apoptotic cell death. By activating AMPK, metformin treatment appears to induce a sensitizing stress that creates a metabolic cellular imbalance in favor of the pro-senescent effects induced by DNA damaging agents. Metformin's ability to accelerate the onset of cellular senescence in HDFs and enhance DNA damage-induced senescence might provide a rational approach to sensitizing pre-malignant and cancer cells to further stress induced by oncogenic stimuli.

### 3. Metformin impedes nuclear reprogramming of somatic cells to induced Pluripotent Stem Cells (iPSCs)

Somatic cells can be reprogrammed by the expression of four factors associated with pluripotency, the so-called “Yamanaka factors” OSKM (O = OCT4, S = SOX2, K = KLF4, M = and c-MYC) [65]. Several groups have observed that a DDR compatible with DNA replication-induced DNA damage is mounted upon the expression of the OSKM reprogramming factors [66-68]. This appears to be similar to what occurs during oncogene-induced senescence (OIS), when cell proliferation and transformation induced by oncogene activation in early tumorigenesis is restrained by cellular senescence, which results from the ATM-mediated DDR triggered by oncogene-induced DNA hyper-replication [69, 70]. However, it should be noted that expression of the four Yamanaka factors has been shown to result in the accumulation of 8-oxoguanine adducts in human fibroblasts, which are commonly the result of oxidative stress. Furthermore, c-MYC overexpression induces DNA damage in a mainly ROS-dependent rather than DNA replication-dependent manner [71, 72]. Therefore, the DNA damage occurring upon reprogramming may be caused not only by OSKM-driven aberrant replication but also through the generation of ROS, which can explain why reprogramming is significantly more efficient under either low oxygen conditions or in the presence of anti-oxidants such as vitamin C [73-76]. Vitamin C efficiently alleviates reprogramming-induced sense-cence (RIS) [66, 75-77], suggesting that antioxidants or other compounds that transiently inhibit senescence could be used to improve reprogramming efficiency. As such, the interplay between the expression of reprogramming factors and the activation of a p53-mediated [68, 78] DDR due to increased DNA replication and/or ROS creates a model in which to test the anti-oxidant (Halicka's findings [39]) or pro-senescent (Vazquez-Martin's findings [12]) effects of metformin in terms of enhanced or repressed reprogramming efficiency, respectively. Because reprogramming in the presence of pre-existing, but tolerated, DNA damage is aborted by the activation of DDR- and p53-dependent apoptosis [68], metformin's ability to reduce ATM activity should attenuate the p53 response to DNA damage (as in some preneoplastic lesions [79, 80]), resulting in accelerated somatic reprogramming. Using MEFs or mouse adult fibroblasts (MAFs), we recently tested the effect of metformin in reprogramming experiments. We found that treatment with metformin dose-dependently inhibited somatic cell reprogramming induced by the OSK stemness factors in MEFs. At 10 mmol/L metformin, iPSC formation was virtually undetectable in MEFs and in MAFs (Vazquez-Martin, Vellon L, Cufi S, Oliveras-Ferraros C, Quirós PM, Lopez-Otin C, Javier A. Menendez. Metformin impedes reprogramming of somatic cells into stem cells. *Manuscript in preparation*). Parallel experiments performed with human BJ-1 fibroblasts transduced with OSKM reprogramming factors produced effects similar to metformin in drastically inhibiting reprogramming (Vazquez-Martin, Vellon L, Cufi S, Oliveras-Ferraros C, Quirós PM, Lopez-Otin C, Javier A. Menendez. Metformin impedes reprogramming of somatic cells into stem cells)*. Manuscript in preparation*). Because p53-mediated DDR limits reprogramming to ensure iPSC genomic integrity [68], it could be argued that these findings are consistent with a genome-protective effect of metformin, which in turn can reduce DNA replication stress [39]. However, metformin exposure was found to abolish highly efficient reprogramming upon abrogation of p53 in MAFs. Importantly, the observed effects on reprogramming efficiencies were not due to metformin-induced cell death of the starting somatic population but rather to the metabolic, pro-senescent effects exerted via AMPK activation [81, 82]. When metformin, which indirectly activates AMPK through effects on the mitochondria, was replaced with the small-molecule A-769662, which directly activates AMPK by mimicking both effects of AMP, including allosteric activation and inhibition of dephosphorylation [83-86], reprogramming efficiency was also drastically reduced.

Recent studies have indicated that somatic cells convert from an oxidative to glycolytic state when they are reprogrammed [87-90] and that the bioenergetic states of somatic cells appear to correlate with their reprogramming efficiencies. Furthermore, manipulating these bioenergetic changes can affect reprogramming, as the glycolysis inhibitor 2-deoxy-D-glucose [2-DG] decreases reprogramming, whereas the glycolysis stimulator D-fructose-6-phosphate (F6P) increases reprogramming of fibroblasts [89, 90]. Although further studies aimed at dissecting the exact mechanism of metformin action in regulating somatic cell reprogramming are needed, it is reasonable to suggest that impaired reprogramming following metformin treatment might result from compromised glycolysis and energy crisis, leading to the sustained activation of AMPK and the establishment of a senescent phenotype, a crucial roadblock for reprogramming [66, 75].

## Metformin and cancer: Hastening the onset of stress-induced senescence to enhance protection against cancer

Understanding the metabolic changes associated with somatic cell reprogramming might shed light on the “metabolic transformation” that is required to support not only the increased biosynthetic needs of the tumor cell but also to enable the acquisition of stemness properties in cancer stem cells (CSCs). Many of the changes in cell metabolism that have been identified to be important in regulating somatic cell reprogramming and induced pluripotency also play roles in oncogenesis. On the other hand, reprogramming to a more dedifferentiated state occurs during tumor progression (i.e., the activation of an embryonic stem cell-like transcriptional program in differentiated adult cells may induce pathologic self-renewal/stemness characteristics of CSCs [91]) and might be favored by alterations in crucial tumor suppressors. Indeed, the stress mechanisms triggered by expression of the Yamanaka stemness factors, which ultimately lead to reprogramming, elicit the tumor suppressor pathways that naturally protect cells against the uncontrolled growth that occurs during tumorigenesis. Unsurprisingly, the cellular response to expression of the reprogramming factors or stem-cell-specific genes molecularly mimics the senescence response observed during OIS, thus emphasizing the parallels between RIS and OIS. In this scenario, if metformin treatment appears to reinforce RIS in somatic reprogramming experiments, we can then infer that metformin treatment should improve cells' ability to establish a more efficient senescence response in pre-malignant and malignant tissues. Many tumor cells appear to have developed mechanisms to reduce AMPK activation and therefore escape from its growth-arrest and tumor-suppressor effects. In fact, more aggressive tumors exhibit reduced signaling via the AMPK pathway, and an inverse relationship exists between the AMPK activation status with histological grade and metastasis [92-94]. As such, metformin-induced energy crisis and, therefore, AMPK (re-)activation, may functionally disrupt the deleterious connection between pluripotency and oncogenic transformation. In fact, Struhl's team discovered that metformin treatment can selectively kill the chemotherapy-resistant subpopulation of CSCs in genetically distinct types of breast cancer cell lines [95, 96]. Our own group has confirmed that treatment with metformin can suppress the self-renewal and proliferation of cancer stem/progenitor cells in *HER2* gene-amplified breast carcinomas cells refractory to HER2-targeted drugs [97, 98]. The central mechanisms through which metformin exposure blocks the ontogenesis of the CSC molecular signature have begun to be elucidated in cultured cancer cells, including alterations of epithelial-to-mesenchymal transition drivers/effectors, tumor-suppressor miRNAs and oncomiRs [99-101]. Metformin's ability to oppose reprogramming of cell energy metabolism from oxidative mitochondria toward an alternative ATP-generating glycolytic metabotype may be sufficient to inhibit the network required for the establishment and maintenance of stem cell pluripotency and self-renewal imposed by certain oncogenic stimuli in the right cellular context [102].

Recently, evidence has emerged that the DDR is one of the earliest events that impedes the multistep progression of human epithelial carcinomas to invasive malignancy. DNA damage can be due to a variety of factors, such as telomere dysfunction or oncogene-induced replication stress [103-105]. Consequently, there is a strong selective pressure for mutation in DDR components because DNA damage checkpoints act as native blockades against the acquisition of invasive/metastatic properties during malignant transformation [103, 105]. Epithelial cells within pre-malignant lesions with markers of senescence maintain an intact response to cellular stress and are less likely to develop subsequent tumors. Accordingly, the presence of functional pro-senescence mechanisms is the most accurate predictor of recurrence and progression of premalignant lesions *in situ* (e.g., in ductal carcinoma in situ [DCIS] of the breast) to biologically aggressive invasive carcinomas (e.g., basal-like breast carcinomas) [106]. Because the DDR is the major innate tumor suppressor barrier in early human tumorigenesis, selective activation of DDR surveillance mechanisms may therefore directly contribute to metformin's cancer preventive effects. Proliferative invasive cancer cells with activated oncogenes acquire mechanisms to suppress senescence in the early stages of cancer pathogenesis (*e.g*., *in situ* lesions). Organisms in which cells fail to undergo senescence die prematurely of cancer [107]. Therefore, activating the program of senescence in tumor cells is an attractive approach to cancer treatment [108, 109] and may help to explain the differential impact of metformin on cancer incidence in non-prone and cancer-prone animal models and perhaps also in cancer-prone individuals. It remains to be clearly defined whether metformin's ability to strongly activate the ATM-regulated DDR checkpoint is the critical event that prevents neoplastic epithelium from progressing unimpeded into invasive cancer in individuals without type 2 diabetes. However, reduced cancer risk in type 2 diabetic patients taking metformin can be explained in terms of metformin's ability to activate DNA damage-like signaling that induces specific senescence-like growth inhibition of pre-malignant or malignant cells without altering the normal function of non-neoplastic tissues. We are currently developing a pre-clinical framework for pro-senescence, metformin-based anti-cancer therapies by evaluating metformin's effects on DCIS xenografts during their spontaneous transition to invasive cancer lesions [110-112]. It would also be relevant to evaluate whether metformin facilitates the “accelerated senescence” triggered in normal cells by the expression of mutated, transforming versions of oncogenes (e.g., *Ras* or *Raf*) and by some other forms of supraphysiological mitogenic signaling irrespective of senescence-inhibiting adaptations (e.g., inactivation of p53) [69, 113, 114]. In a clinical scenario, it would be interesting to test whether metformin can significantly increase senescence in premalignant lesions of the skin, the lung, the pancreas, the liver or the breast. Additionally, forthcoming studies should evaluate metformin's effects in clinical scenarios in which senescence has been recognized to have positive effects on organ maintenance. Senescence limits pathological responses to either acute forms of injury such as fibrotic scarring in response to chemically induced liver injury [98, 115, 116] or to chronic viral infections such as hepatitis C virus (HCV) with or without concomitant human immunodeficiency virus (HIV) infection. Indeed, HCV infection increases rates of hepatocellular carcinoma via the accumulation of senescent hepatocytes in human liver [117].

## Metformin: Lowering the threshold for stress-induced senescence to limit cancer development and delay aging-associated disorders

Metformin's ability to enhance senescence in established premalignant disease or in fully malignant disease is a largely unexplored mechanism that may explain why reductions in cancer mortality related to metformin use are similar in magnitude to reductions in cancer incidence. This suggests that the anti-cancer effects of metformin largely depend on (or are restricted to) its preventive effects [118]. The most widely accepted interpretation for the biological function of cellular senescence is that it serves as a mechanism for restricting cancer progression. Based on this, escaping from cellular senescence and becoming immortal constitute a required additional step in the progression of oncogenesis [51, 52]. Recent studies have suggested that the accumulation of ROS and oxidative damage are commonly involved in culture stress- or oncogene-induced cellular senescence. Increasing accumulation of ROS is observed during replicative senescence, and the replicative potential of MEFs and HDFs is significantly higher under low oxygen conditions. As such, the ability of immortalized cells, including embryonic stem cells (ESCs), iPSCs and CSCs, to buffer oxidative stress may be pivotal for explaining their immortality [53-56; 87-90]. Enhanced glycolysis actively protects cells from senescence induced by oxidative stress [53-56], a metabolic protection that appears to causally contribute to the maintenance of the self-renewal capacity of stem cells [87-90]. In fact, the enhanced glycolysis of the Warburg effect is a crucial metabolic feature that helps cancer cells bypass senescence, and this may provide indirect evidence that metformin's primary target is the immortalizing step during tumorigenesis. In other words, if enhanced glycolysis is necessary and sufficient to enable indefinite proliferation (i.e., immortalization) very early during multi-step carcinogenesis *in vivo*, then metformin's ability to inhibit glucose flux while simultaneously stimulating lactate/pyruvate flux and mitochondrial biogenesis must cause ATP depletion accompanied by a drastic increase in cellular AMP, which is expected to induce premature senescence [50]. Many tumor cells retain the ability to senesce in response to DNA-damaging drugs in culture and *in vivo*. Because of this, metformin-accelerated replicative senescence due to a stronger DDR-dependent cell cycle arrest may underlie metformin's ability to increase the rate of pathological complete response (pCR) in neoadjuvant chemotherapy in diabetic patients with breast cancer [119] and to promote tumor regression and prevent relapse when combined with suboptimal doses of chemotherapy in animal models [96].

Senescent cells accumulate in various tissues and organs with aging and have been hypothesized to disrupt tissue structure and function [120-122]. Cellular senescence halts the proliferation of damaged or dysfunctional cells, thus functioning as a pivotal mechanism to constrain the malignant progression of tumor cells [123, 124]. Indeed, upon the aberrant activation of oncogenes, normal cells can enter the cellular senescence program, a state of stable cell-cycle arrest that represents an important barrier against tumor development *in vivo* [125]. Senescent cells communicate with their environment by secreting various cytokines and growth factors, and this “secretory phenotype” has been reported to have pro- as well as anti-tumorigenic effects [126-130]. In this regard, it is remarkable that metformin-induced chronic activation of ATM signaling in A431 epidermoid cancer cells is accompanied by the increased expression of a wide variety of cyto-/chemokines (e.g., IL6, IL1B, CCL3, CCL5, IL1F9 or CXCL11), as measured by Agilent's whole human genome arrays (Figure 3C) (Cufí S, Vazquez-Martin A, Oliveras-Ferraros C, Martin-Castillo B, Vellon L, Menendez JA. Metformin lowers the threshold for stress-induced cellular senescence. *Manuscript in preparation*). Cellular senescence is often the result of ATM sensing of nuclear DNA damage fueling chronic DDR, and upstream elements of the DDR signaling cascade are necessary to successfully establish a SASP [131]. Thus, it might be tempting to suggest that an AMPK-induced energy crisis imposed by continuous exposure to metformin is a novel regulator of the DDR-SASP axis that reinforces the senescent phenotype.

Two recent landmark studies [117, 132] help clarify the apparently counterintuitive pro-senescent activity of the anti-aging biguanide metformin [133-138]. Kang et al. [117] have confirmed that pre-malignant senescent hepatocytes secrete chemo- and cytokines and are subject to immune-mediated clearance (“senescence surveillance”) that strictly depends on an intact CD4+ T-cell-mediated adaptive immune response. Accordingly, impaired immune surveillance of pre-malignant senescent hepatocytes results in the development of hepatocellular carcinomas, thus showing that senescence surveillance is important for tumor suppression *in vivo* by mounting specific immune responses against antigens expressed in pre-malignant senescent cells [117]. The molecular mechanisms presented here suggest that metformin's use of the senescence program may significantly diminish the number of cells that can escape senescence. By activating the chemopreventive ATM-dependent DDR, metformin-treated cells might be sensitized for damage signaling, and metformin treatment might mimic the precancerous stimulus that generates a barrier against carcinogenesis. Because energy metabolism is being recognized as a/the critical pathway regulating the immortalization-to-senescence transition, metformin-based therapies based on the induction of senescence would prevent the generation of CSCs. Concurrently, metformin treatment could lead to a significant alteration of “cancer immunoediting” (e.g., via restoration of MHC-related pathways) to impede tumor cell escape from immunosurveillance. The net effect of metformin treatment should be, therefore, a significant decrease in the accumulation of dysfunctional, pre-malignant cells in tissues, including those with the ability to initiate tumors (i.e., CSCs).

Whether senescent cells are causally implicated in age-related dysfunction and whether their removal is beneficial have remained unknown until now. Baker et al. [132] have nicely answered this pivotal question by designing a transgenic strategy for the clearance of senescent cells in mice. Using a BubR1 progeroid mouse background designed for inducible elimination of p16^Ink4a^-positive senescent cells, the authors demonstrate that in tissues in which p16^Ink4a^ contributes to the acquisition of age-related pathologies (i.e., adipose tissue, skeletal muscle and eye), life-long removal of p16^Ink4a^-expressing cells significantly delays onset of these pathologies. Furthermore, late-life clearance notably attenuates progression of already established age-related disorders [132]. If metformin's ability to prevent escape from the senescence anti-tumor barrier is accompanied by an enhanced clearance of pre-malignant/tumor senescent cells, including those exhibiting tumor-initiating ability (i.e., CSCs), the net effect of the tissue sweeper functioning of metformin will be to halt the malignant/metastatic progression of pre-malignant/tumor senescent cells while preventing or delaying tissue dysfunction, thus extending the human lifespan.

## Note

In addition to SA-β-gal activity, senescence has been previously linked to induction of ROS production, which is believed to be necessary for maintenance of the senescence phenotype [139, 140]. ROS also play a pivotal role in promoting SA-β-gal activity following radiation-induced DNA damage. Accordingly, inhibition of ROS using N-acetyl cysteine (NAC) following radiation treatment drastically decreases senescence in tumor cells with normally high levels of radiation-induced SA-β-gal. In accordance with our current observations and taking advantage of earlier studies suggesting that metformin treatment induces ROS in certain cellular backgrounds [141], Skinner et al [142] have recently confirmed that enhanced rather than reduced ROS and SA-β-gal activity occurred when metformin was concurrently added to radiation in p53-deficient tumor cells. Skinner's findings that metformin treatment can overcome locoregional treatment failure in head and neck carcinomas by reinforcing the radiation-induced cellular senescence, which clinically translates into a significantly improved survival in patients taking metformin, strongly support our hypothesis that metformin's ability to reinforce the establishment of accelerated senescence may function as an effective barrier to tumor growth and disease recurrence.
